# Comparative Efficacy and Safety of Mycophenolate Mofetil Versus Cyclophosphamide As Induction Therapy for Lupus Nephritis: A Systematic Review and Meta-Analysis

**DOI:** 10.7759/cureus.111741

**Published:** 2026-06-29

**Authors:** Basel Mousa, Nedal H Elrewany, Ahmad Abdelnasser A Noor, Omar Tubaileh, Ahmed Silman, Natalie Fayez Kobal, Saman Akhtar, Faiqa Tariq, Sheeza Nadeem, Hanin Abdelrahman Khalaf

**Affiliations:** 1 Internal Medicine, Kharkiv National Medical University, Kharkiv, UKR; 2 Internal Medicine, Royal College of Surgeons in Ireland - Medical University of Bahrain, Busaiteen, BHR; 3 Internal Medicine, Eras Lucknow Medical College and Hospital, Lucknow, IND; 4 Acute Medicine, Scunthorpe General Hospital, Scunthorpe, GBR; 5 General Internal Medicine, Jinnah Medical & Dental College, Karachi, PAK; 6 Internal Medicine, Doncaster and Bassetlaw Teaching Hospitals NHS Foundation Trust, Doncaster, GBR; 7 Obstetrics and Gynecology, Beirut Arab University, Beirut, LBN

**Keywords:** cyclophosphamide, induction therapy, lupus nephritis, meta-analysis, mycophenolate mofetil, systematic review

## Abstract

Lupus nephritis (LN) is a serious and potentially fatal systemic lupus erythematosus complication that causes substantial morbidity and may progress to end-stage kidney disease. Traditionally, cyclophosphamide (CYC) has been prescribed as induction therapy for LN. However, there has been a trend toward using mycophenolate mofetil (MMF) as an alternative treatment due to concerns of toxicity. This systematic review and meta-analysis evaluated the comparative safety and efficacy of MMF to CYC as induction therapy in LN. PubMed, Embase, Scopus, and Cochrane CENTRAL databases were searched comprehensively from their inception through January 2026. Randomized controlled trials and observational studies comparing MMF and CYC in adult or pediatric patients with LN were included. Complete renal remission, partial renal remission, overall renal response, mortality, infections, GI adverse events, leukopenia, menstrual disorders, and alopecia were evaluated as outcomes. Overall, 12 studies involving 1,502 patients were analyzed. MMF was significantly associated with a higher rate of complete renal remission than CYC (risk ratio (RR) = 1.11, 95% confidence interval (CI): 1.01-1.22). No significant difference between the two therapies in partial renal remission, total renal response, mortality, infection, GI adverse events, or leukopenia. However, MMF was associated with significantly lower risks of menstrual disorders (RR = 0.47, 95% CI: 0.27-0.82) and alopecia (RR = 0.32, 95% CI: 0.15-0.70). Overall, MMF appears to be as effective as CYC for the induction treatment of LN, while exhibiting a more favorable safety profile regarding reproductive and cosmetic adverse events. The results indicate that MMF is an effective and safer induction agent for patients with LN.

## Introduction and background

Lupus nephritis (LN) is a serious and clinically important complication of systemic lupus erythematosus that occurs in about 30-60% of patients throughout the course of the disease [1]. LN is an autoimmune inflammatory disease of the kidneys followed by progressive tissue injury, which can result in chronic kidney damage, end-stage renal disease, and higher morbidity and mortality if not treated properly [2,3]. Despite the progress made in therapeutic approaches, LN still carries a heavy burden and places substantial strain on healthcare systems, markedly affecting patients' quality of life, especially for young girls, who constitute the majority of affected patients [4,5]. LN is classified histologically according to the International Society of Nephrology/Renal Pathology Society classification system. Class III and IV represent proliferative LN, whereas Class V represents membranous LN. Active Class III/IV disease and Class V disease with significant proteinuria commonly require induction immunosuppressive therapy [6,7].

Typical treatment for LN includes induction and maintenance therapy, with induction therapy aimed at quickly controlling active inflammation in the kidneys [8]. Cyclophosphamide (CYC) has long been used as a standard induction agent due to its strong immunosuppressive properties and its effectiveness in severe disease [9]. However, its use is linked with considerable adverse events, including infections, infertility, ovarian failure, malignancy risk, and hematological toxicity [10]. Mycophenolate mofetil (MMF), a selective inhibitor of inosine monophosphate dehydrogenase that suppresses T- and B-lymphocyte proliferation, has emerged as a promising alternative induction agent. Contemporary treatment recommendations and clinical studies suggest that MMF is as effective as CYC while offering improved tolerability [1,11,12].

Several systematic reviews and meta-analyses have compared MMF and CYC as induction therapy for LN. Earlier evidence syntheses by Kamanamool et al., Liu et al., Henderson et al., Palmer et al., and Jiang et al. consistently reported comparable overall renal response rates between the two therapies while suggesting a more favorable safety profile for MMF [13-17]. However, these reviews were conducted before the publication of several recent comparative studies and were largely based on adult populations. Additionally, outcome reporting varied considerably across studies, and important safety outcomes such as menstrual disorders and alopecia were not consistently evaluated. More recently, Sarkar et al. performed a meta-analysis focused exclusively on childhood-onset LN, limiting the applicability of its findings to the broader LN population [18]. Therefore, an updated systematic review and meta-analysis incorporating both adult and pediatric populations, recently published evidence, and a comprehensive assessment of efficacy and safety outcomes is warranted. The present study aims to provide a contemporary synthesis of the available evidence and determine whether the inclusion of newer studies alters previous estimates of treatment efficacy and safety, thereby offering more robust guidance for clinical decision-making.

## Review

To guarantee transparency, reproducibility, and methodological rigor, this systematic review and meta-analysis were conducted in accordance with the Preferred Reporting Items for Systematic Reviews and Meta-Analyses (PRISMA 2020) criteria. Before data collection and statistical analysis, the review methodology was prospectively registered with the International Prospective Register of Systematic Reviews (PROSPERO) (CRD420261396218) [19].

Data source and search strategy

A thorough and methodical search of the literature was conducted in PubMed, Embase, Scopus, and the Cochrane Central Register of Controlled Trials (CENTRAL) from database inception to January 2026, with no time constraints. Included were only English-language studies.

Medical Subject Headings (MeSH) and free-text keywords associated with LN and the relevant interventions were combined in the search approach. "Mycophenolate mofetil", "MMF", "cyclophosphamide", "CYC", "lupus nephritis", "systemic lupus erythematosus nephritis", and "SLE nephritis" were among the search phrases used. When appropriate, other methodological words such as "randomized controlled trial", "cohort study", "observational study", and "clinical trial" were also employed. To increase the sensitivity and specificity of the search, Boolean operators (AND/OR) were used. To identify additional studies, the reference lists of eligible papers, earlier reviews, and meta-analyses were manually searched, in addition to electronic database searches. Supplementary Appendix 1 outlines a thorough search strategy for each database. Eligibility criteria were determined using the population, intervention, comparator, and outcomes (PICO) framework (Tables 1-2).

**Table 1:** Inclusion criteria for study selection based on the PICO framework MMF: mycophenolate mofetil, CYC: cyclophosphamide, RCTs: randomized controlled trials, PICO: population, intervention, comparator, and outcomes, GI: gastrointestinal, LN: lupus nephritis

Domain	Inclusion criteria
Population	Adult and pediatric patients with biopsy-proven or clinically diagnosed LN according to established classification criteria.
Intervention	MMF administered as induction therapy at any approved or study-defined dosage and duration.
Comparator	CYC-based induction therapy administered orally or intravenously according to the study protocol.
Outcomes	Studies reporting at least one efficacy or safety outcome, including complete renal remission, partial renal remission, total renal response, relapse, adverse events, infection, leukopenia, GI adverse events, amenorrhea, or mortality.
Study design	RCTs, prospective cohort studies, and retrospective cohort studies directly comparing MMF and CYC as induction therapy for LN.
Data availability	Studies providing sufficient numerical data to calculate effect estimates (e.g., event counts, odds ratios, risk ratios, or corresponding raw data).

**Table 2:** Exclusion criteria for study selection based on the PICO framework MMF: mycophenolate mofetil, CYC: cyclophosphamide, PICO: population, intervention, comparator, and outcomes

Criterion	Description
Non-comparative studies	Studies not directly comparing MMF and CYC as induction therapy.
Maintenance therapy studies	Studies evaluating maintenance therapy only or mixed treatment regimens without separately extractable MMF and CYC induction data.
Publication type	Case reports, case series, editorials, letters, narrative reviews, systematic reviews, and meta-analyses.
Insufficient reporting	Conference abstracts, meeting proceedings, and unpublished reports lacking sufficient extractable data.
Preclinical studies	Animal, in vitro, or other laboratory-based investigations.
Duplicate populations	Duplicate publications or studies reporting overlapping patient populations; the most comprehensive or recent report was retained.
Insufficient statistical data	Studies lacking adequate numerical information to calculate effect estimates.

Screening and study selection process

Two reviewers individually screened all identified studies in two steps. Titles and abstracts were first assessed for relevance, followed by a full-text review of potentially eligible studies. Only studies that met all inclusion criteria were included in the final qualitative and quantitative syntheses. Disagreements over study selection were handled by debate or arbitration by a third reviewer. The number of recognized, screened, excluded, and included studies was recorded in the PRISMA flow diagram.

Data extraction

To minimize errors and inconsistencies, two reviewers independently extracted data using a standardized data extraction form. The following information was collected from each study: (1) study characteristics, including author, publication year, country, and study design; (2) population characteristics, including sample size, age group, pediatric or adult population, and LN classification; (3) intervention details, including MMF dosage and treatment duration; (4) comparator regimen, including CYC protocol and duration; (5) efficacy outcomes, including complete renal remission, partial renal remission, and overall renal response; and (6) safety outcomes, including adverse events, infections, leukopenia, GI adverse events, menstrual disorders, and mortality. Risk ratios (RRs) with 95% confidence intervals (CIs) were reported for dichotomous outcomes. Any disagreements were resolved through discussion or consultation with a third reviewer.

Risk of bias assessment for randomized controlled trials

**Figure 1:**
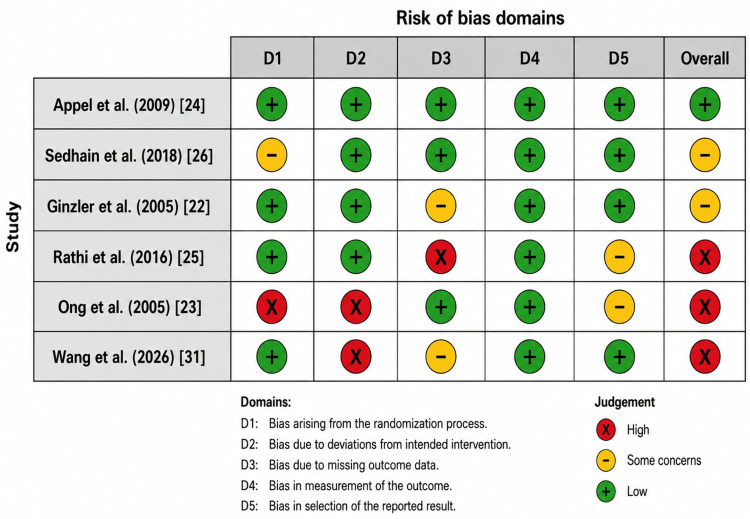
Risk of bias assessment of included studies in domains Risk of bias was assessed across five domains, including randomization process (D1), deviations from intended interventions (D2), missing outcome data (D3), outcome measurement (D4), and selective reporting (D5).

**Figure 2:**
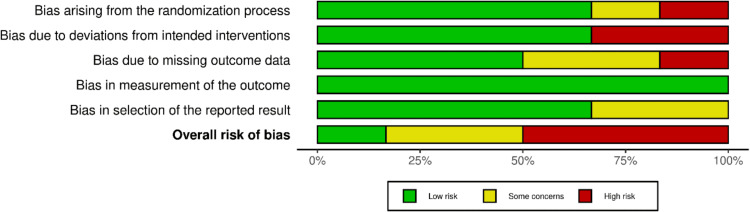
Traffic plots of included studies Traffic plot showing the overall risk-of-bias judgment for each study.

Two reviewers independently assessed the methodological quality of the included randomized controlled trials (RCTs) using the Risk of Bias 2.0 tool (RoB 2; Cochrane, London, UK). Six RCTs were assessed across five methodological domains: bias from the randomization process, bias due to deviations from the intended intervention, bias due to missing outcome data, bias in outcome assessment, and bias in the selection of reported results. The RoB 2 guiding criteria were used to classify each domain as "low risk," "some concerns," or "high risk" of bias. The overall risk-of-bias rating for each trial was then determined by assigning the highest level of risk of bias observed across the various domains. Disagreements between reviewers were addressed via discussion and consensus, or if necessary, by consulting a third reviewer [20]. The results of the evaluations were presented as traffic-light and summary graphs (Figures 1-2).

Newcastle-Ottawa Scale for observational studies

The methodological quality of the included observational studies was evaluated separately by two reviewers using the Newcastle-Ottawa Scale (NOS). Six observational studies were examined in three primary domains: group selection, group comparability, and outcome assessment. The NOS values ranged from 0 to 9, with higher numbers indicating better methodological quality and a lower risk of bias. Studies with higher ratings were thought to have more methodological rigor and internal validity. Any conflicts in ratings between reviewers were resolved through discussion and consensus, with assistance from a third reviewer as needed [21].

Statistical analysis

Statistical analyses were carried out using Review Manager (RevMan) version 5.4.1 (The Cochrane Collaboration, Copenhagen, Denmark). The Mantel-Haenszel random-effects model was used to pool dichotomous outcomes, such as total renal remission, partial renal remission, overall renal response, and adverse events, and the results were reported as RRs with 95% CIs.

The Chi-square test and the I² statistic were used to assess and quantify statistical heterogeneity among studies. An I² value above 50% indicated significant heterogeneity. A random-effects model was employed to account for clinical and methodological diversity between studies.

Sensitivity analyses were performed where needed to assess the robustness of aggregated findings. For outcomes with sufficient numbers of included papers, publication bias was examined visually using funnel plot analysis. A two-sided P value of less than 0.05 was considered statistically significant.

Results

**Figure 3:**
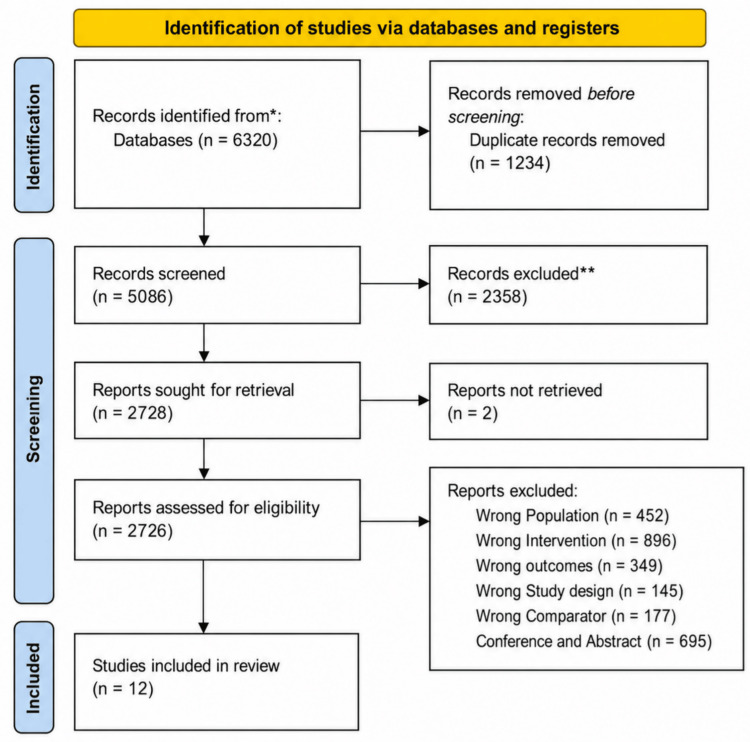
PRISMA flow diagram of included studies A total of 6,320 records were identified from databases. After removing 1,234 duplicates, 5,086 records were screened, and 2,358 were excluded at the title/abstract level. Following full-text assessment of 2,726 reports, 2,714 were excluded for reasons including wrong population, intervention, outcomes, study design, comparator, or conference abstracts, resulting in 12 studies finally included in the systematic review and meta-analysis. PRISMA: Preferred Reporting Items for Systematic Reviews and Meta-Analyses

From an initial identification of 6,320 records in databases, 1,234 duplicates were deleted, leaving 5,086 records for screening. After removing 2,358 records at the title/abstract stage, 2,728 reports were sought for retrieval; however, 2 were not found. Following a full-text evaluation of 2,726 papers, 2,714 were removed for reasons such as incorrect population, intervention, outcomes, research design, comparator, or conference abstracts, leaving just 12 studies in the analysis (Figure 3).

Baseline characteristics and patient demographics

The included studies comprised RCTs and observational studies conducted in the United States, Malaysia, India, Nepal, China, Thailand, the United Kingdom, France, and the Philippines. The majority of participants were young females, with female representation ranging from 76% to 96.5%. The mean age in adult cohorts ranged from 24.6 to 39 years, whereas pediatric trials showed mean ages of 12-13 years. The majority of patients had proliferative LN, mainly Class IV illness (52-76%), while Class III and V nephritis were also seen in various studies. Baseline renal function was generally normal to modestly impaired, with serum creatinine levels ranging from 0.71 to 1.73 mg/dL and pediatric studies finding eGFR values of 92-102 mL/min. Baseline proteinuria suggested active disease severity, with urine protein excretion typically ranging from 1.8 to 5.1 g/day and increased uPCR/uACR levels across the trials examined (Table 3).

**Table 3:** Characteristics and baseline demographics of included studies Baseline characteristics and demographics of 12 included studies (six RCTs, six observational; N = 1,849 patients). Groups were well-balanced, with female predominance (76-96%), mean age 12-39 years, Class III-IV LN, proteinuria 1.8-5.1 g/24 h, and serum creatinine 0.71-1.73 mg/dL. MMF: mycophenolate mofetil, CYC: cyclophosphamide, RCT: randomized controlled trial, Obs: observational study, retro: retrospective, USA: United States, UK: United Kingdom, uPCR: urine protein-to-creatinine ratio, uACR: urine albumin-to-creatinine ratio, eGFR: estimated glomerular filtration rate, LN: lupus nephritis

Study ID (author, year)	Study design	Population/country	Sample size (MMF/CYC)	Age (years) (mean/med)	Gender (%female)	Baseline renal class (III/IV/V)	Baseline serum creatinine (mg/dL)	Baseline proteinuria (g/24 h or ratio)
Ginzler et al., 2005 [22]	RCT	Adult/USA	71/69	31.0-32.5	86-94%	15% III, 55% IV, 20% V	1.06-1.08	4.1-4.4 g
Ong et al., 2005 [23]	RCT	Adult/Malaysia	19/25	30.5-31.3	79-88%	5.3% III, 52.6% IV	~1.08 (95 μmol/L)	1.8-3.0 g
Appel et al., 2009 [24]	RCT	Adult/global	185/185	31.3-32.4	84.6%	15.7% III, 68.1% IV, 16.2% V	1.04-1.22	4.1 (uPCR)
Rathi et al., 2016 [25]	RCT	Adult/India	50/50	28.3-30.6	90-94%	12-22% III, 54-60% IV	~0.88 (78 μmol/L)	2.2-3.0 (uPCR)
Sedhain et al., 2018 [26]	RCT	Adult/Nepal	21/21	24.6-27.2	85.7-90.5%	19-23% III, 62-76% IV	1.24-1.73	3.3 g
Smith et al., 2019 [27]	Obs (retro)	Pediatric/UK	34 / 17	12.6-13.3	79-82%	III or IV	~0.71 (63 μmol/L)	45-177 (uACR)
Prasad et al., 2020 [28]	Obs (retro)	Adult/India	33/67	31.6 ± 11.5	94%	37% III, 38% IV	1.21-1.54	4.6-5.1 g
Chbihi et al., 2022 [29]	Obs (retro)	Pediatric/France	17/16	12.5-13.1	76-88%	IV	~0.85 (75 μmol/L)	272-474 (uPCR)
Zhang et al., 2023 [12]	Obs (real-world)	Adult/China	98/97	31.4-39.0	83.6%	III-V	~0.85 (76 μmol/L)	3.1-3.6 g
Poungsuwan et al., 2025 [30]	Obs (retro)	Adult/Thailand	34/55	33.9-35.9	78.2-82.4%	III-V	1.1-1.5	3.8-4.1 (uPCR)
Wang et al., 2026 [31]	RCT	Pediatric/China	52/55	~12.0	77-78%	3/4 ± 5	eGFR 92-102 ml/min	2.8-3.0 g
Guiang-Valerio et al., 2026 [32]	Obs (retro)	Pediatric/Philippines	57/174	12.5-13.0	92.5-96.5%	III-V	0.79-0.83	2.05-3.88 (uPCR)

Quality assessment

Based on the risk-of-bias assessment, the included studies had varying methodological quality. Appel et al. [24] had low risk across all categories; however, Sedhain et al. [26] and Ginzler et al. [22] had high risk due to randomization and missing outcome data, respectively. Rathi et al. [25], Ong et al. [23], and Wang et al. [31] raised concerns across a variety of disciplines, notably regarding the selection of reported outcomes and missing outcome data. Overall, only one study had a low risk of bias, two had a high risk, and three raised some concerns, demonstrating substantial methodological heterogeneity across trials comparing MMF with CYC for induction therapy in LN (Figures 1-2). Overall, the five observational studies had good methodological quality according to the NOS. Three studies (Smith et al. [27], Chbihi et al. [29], and Zhang et al. [12]) received the highest rating of nine stars for acceptable representativeness, exposure ascertainment, and comparability while controlling for confounders. The remaining three investigations (Prasad et al. [28], Poungsuwan et al. [30], and Guiang-Valerio et al. [32]) received eight stars each, largely due to missing points for comparability or follow-up adequacy (Table 4).

**Table 4:** Quality assessment of included observational studies using the NOS The total score reflects the overall quality assessment of each study. A score of 9 indicates high quality, scores of 6-8 indicate good quality, and scores below 6 indicate low quality. **☆: adherence to the criteria. Numbers in parentheses () represent the maximum possible score for each criterion. NOS: Newcastle-Ottawa Scale

Study ID	Representativeness of the exposed cohort (1)	Selection of the non-exposed cohort (1)	Ascertainment of exposure (1)	Demonstration that the outcome of interest was not present at the start of the study (1)	Comparability of cohorts based on the design or analysis controlled for confounders (2)	Assessment of outcome (1)	Was the follow-up long enough for outcomes to occur (1)	Adequacy of follow-up of cohorts (1)	Total score (9)
Smith et al. (2019) [27]	★	★	★	★	★★	★	★	★	9
Chbihi et al. (2022) [29]	★	★	★	★	★★	★	★	★	9
Zhang et al. (2023) [12]	★	★	★	★	★★	★	★	★	9
Prasad et al. (2020) [28]	★	★	★	★	★	★	★	★	8
Poungsuwan et al. (2025) [30]	★	★	★	★	★	★	★	★	8
Guiang-Valerio et al. (2026) [32]	★	★	★	★	★	★	★	★	8

Outcomes

Efficacy and safety outcomes are summarized in Table 5.

**Table 5:** Renal efficacy and safety outcomes comparing MMF and CYC for the treatment of LN Renal efficacy and safety outcomes comparing MMF versus CYC across all studies. Total renal response rates were comparable between groups, whereas MMF was associated with significantly lower rates of alopecia and menstrual disorders than CYC. MMF: mycophenolate mofetil, CYC: cyclophosphamide, LN: lupus nephritis

Author, year	Complete renal remission (MMF vs CYC) event/total	Partial renal remission (MMF vs CYC) event/total	Total renal response (MMF vs CYC) event/total	Patient death (MMF vs CYC) event/total	Infections (MMF vs CYC) event/total	GI adverse events (MMF vs CYC) event/total	Leucopenia (MMF vs CYC) event/total	Menstrual disorders (MMF vs CYC) event/total	Alopecia (MMF vs CYC) event/total
Ginzler et al., 2005 [22]	16/71 vs 4/69	21/71 vs 17/69	37/71 vs 21/69	0/71 vs 3/69	27/83 vs 29/75	38/83 vs 27/75	18/83 vs 28/75	8/83 vs 13/75	0/83 vs 8/75
Ong et al., 2005 [23]	5/19 vs 3/25	6/19 vs 10/25	11/19 vs 13/25	1/19 vs 1/25	3/19 vs 3/25	N/A	7/19 vs 13/25	0/19 vs 1/25	N/A
Appel et al., 2009 [24]	16/185 vs 15/185	88/185 vs 83/185	104/185 vs 98/185	9/185 vs 5/185	126/184 vs 111/180	113/184 vs 120/180	N/A	N/A	20/184 vs 64/180
Rathi et al., 2016 [25]	27/50 vs 25/50	10/50 vs 12/50	37/50 vs 37/50	5/50 vs 2/50	10/50 vs 13/50	26/50 vs 2/50	7/50 vs 5/50	2/50 vs 1/50	0/50 vs 1/50
Sedhain et al., 2018 [26]	14/21 vs 14/21	4/21 vs 4/21	18/21 vs 18/21	0/21 vs 0/21	7/21 vs 10/21	0/21 vs 16/21	N/A	N/A	0/21 vs 16/21
Prasad et al., 2020 [28]	19/33 vs 31/67	4/33 vs 16/67	23/33 vs 47/67	3/33 vs 10/67	4/33 vs 14/67	11/33 vs 9/67	3/33 vs 5/67	2/30 vs 15/64	2/33 vs 10/67
Chbihi et al., 2022 [29]	9/17 vs 11/16	1/17 vs 0/16	10/17 vs 11/16	0/17 vs 0/16	3/17 vs 3/16	2/17 vs 0/16	2/17 vs 0/16	N/A	N/A
Zhang et al., 2023 [12]	46/97 vs 35/94	31/97 vs 25/94	77/97 vs 60/94	0/98 vs 0/97	29/98 vs 39/97	2/98 vs 16/97	2/98 vs 4/97	4/98 vs 11/97	6/98 vs 5/97
Poungsuwan et al., 2025 [30]	15/34 vs 22/55	14/34 vs 23/55	29/34 vs 45/55	0/34 vs 1/55	2/34 vs 6/55	3/34 vs 1/55	0/34 vs 4/55	0/34 vs 1/55	0/34 vs 4/55
Wang et al., 2026 [31]	40/52 vs 38/55	5/52 vs 7/55	48/52 vs 49/55	N/A	4/52 vs 4/55	0/52 vs 0/55	0/52 vs 1/55	0/52 vs 1/55	N/A
Guiang-Valerio, 2026 [32]	48/57 vs 134/174	5/57 vs 30/174	53/57 vs 164/174	0/57 vs 0/174	N/A vs 19/174	N/A	N/A	N/A	N/A
Smith et al., 2019 [27]	23/32 vs 11/15	3/32 vs 2/15	26/32 vs 13/15	N/A	N/A	N/A	N/A	N/A	N/A

Efficacy Outcomes

**Figure 4:**
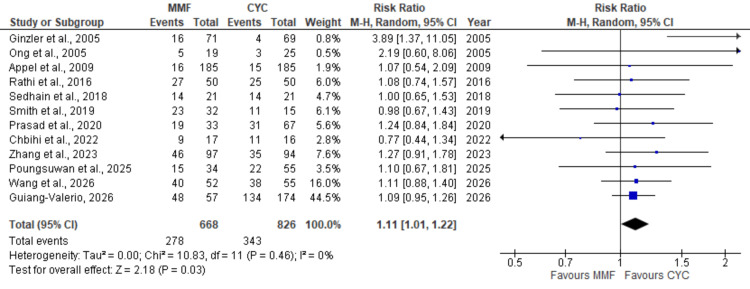
Forest plot of complete renal remission MMF: mycophenolate mofetil, CYC: cyclophosphamide, CI: confidence interval

**Figure 5:**
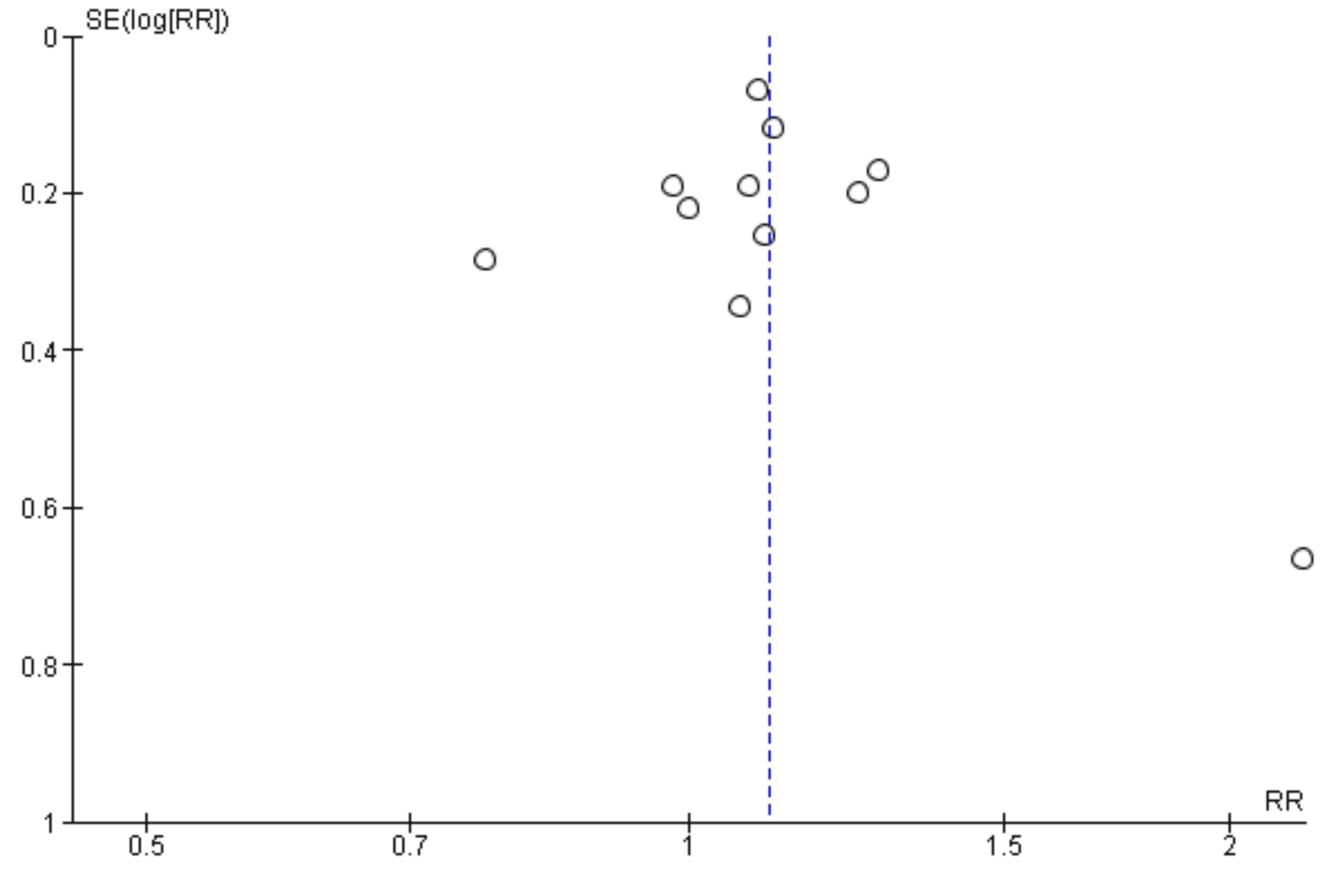
Funnel plot assessing publication bias for complete renal remission

Complete renal remission: A pooled analysis of 12 studies [12,22-32] involving 1,494 patients (668 treated with MMF and 826 treated with CYC) evaluated complete renal remission in patients with LN. The pooled analysis of CYC was associated with a significantly lower rate of complete renal remission when compared to MMF (RR = 1.11, 95% CI: 1.01-1.22, P = 0.03). There was no statistical heterogeneity among studies (P = 0.46; I² = 0%), suggesting high consistency of findings across the included trials (Figure 4). Furthermore, the funnel plot for complete renal remission showed quite a symmetrical distribution of studies around the pooled effect estimate, indicating the absence of significant publication bias. One study was somewhat removed from the other studies. Still, the others' pattern remained balanced, indicating the strength and reliability of the group's conclusions (Figure 5).

**Figure 6:**
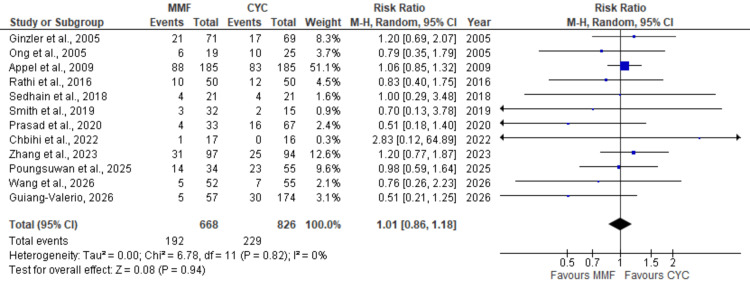
Forest plot of partial remission response MMF: mycophenolate mofetil, CYC: cyclophosphamide, CI: confidence interval

**Figure 7:**
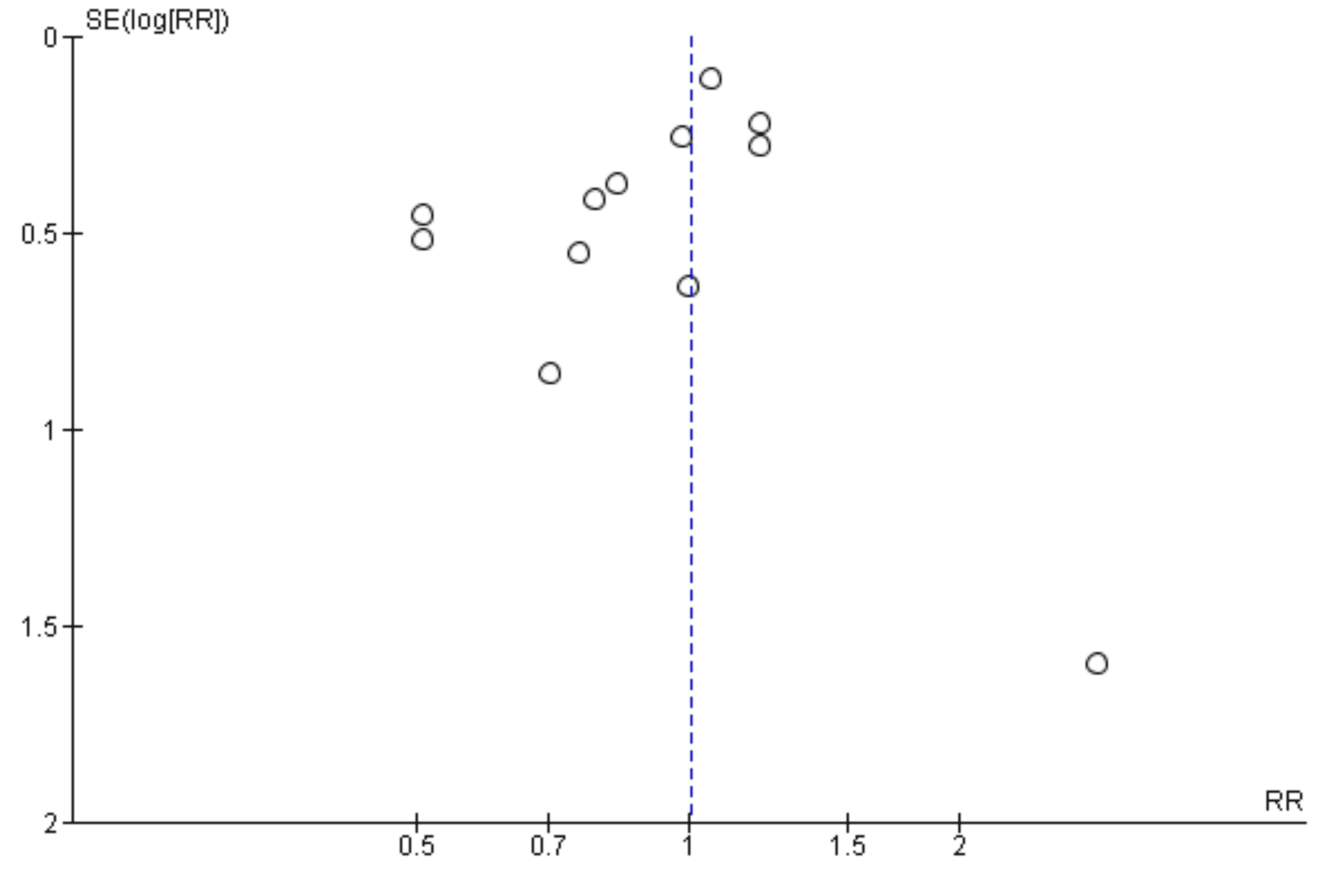
Funnel plot assessing publication bias for partial renal remission

Partial remission response: The 12 studies [12,22-32] were included in the pooled analysis for partial renal remission and comprised 1494 participants. There was no significant difference between MMF and CYC in achieving partial renal remission (RR = 1.01, 95% CI: 0.86-1.18, P = 0.94). The difference between the results of the separate studies was not statistically significant (P = 0.82; I² = 0%), indicating uniformity across the studies (Figure 6). Plotting the funnel indicated a fairly even distribution of studies around the pooled effect size, with slight scatter in the smaller studies. This indicates that there is no publication bias or small-study effect among the included studies (Figure 7).

**Figure 8:**
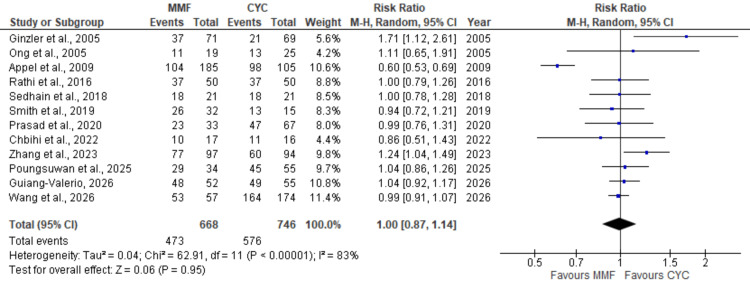
Forest plot of total remission response MMF: mycophenolate mofetil, CYC: cyclophosphamide, CI: confidence interval

**Figure 9:**
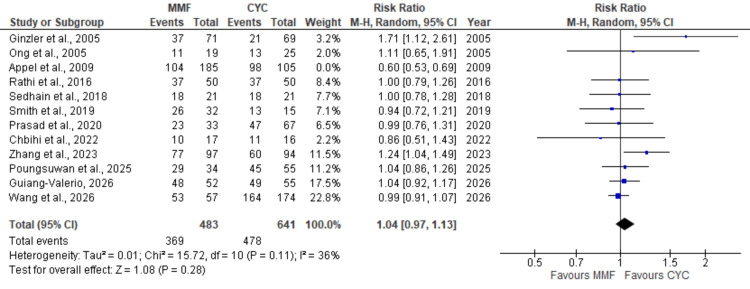
Sensitivity analysis of total remission response MMF: mycophenolate mofetil, CYC: cyclophosphamide, CI: confidence interval

**Figure 10:**
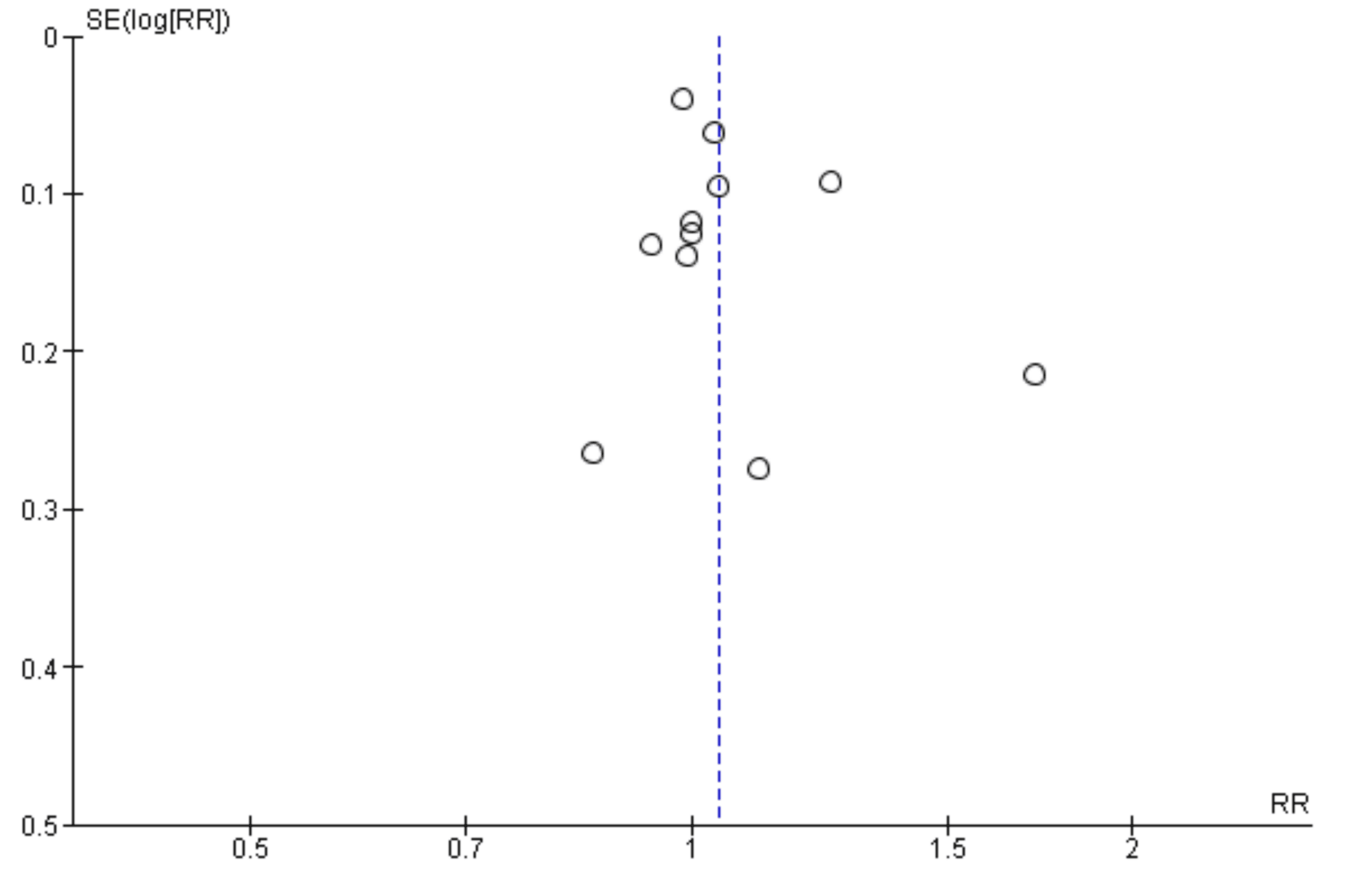
Funnel plot assessing publication bias for total renal remission

Total remission response: The analysis of complete renal remission included 12 studies [12,22-32], with 668 patients in the MMF group and 746 in the CYC group. The pooled RR for complete renal remission was 1.00 (95% CI: 0.87-1.14; P = 0.95), which was not statistically significant compared with CYC. The degree of heterogeneity between the studies was high (I² = 83, P < 0.00001), indicating significant differences among the included studies (Figure 8). To assess the possibility of substantial heterogeneity in the initial pooled analysis, a sensitivity analysis was conducted by excluding the study that contributed most to the heterogeneity. After exclusion, a total of 11 studies, comprising 483 patients in the MMF group and 641 in the CYC group, were included in the analysis. The results of the sensitivity analysis revealed a pooled RR of 1.04 (95% CI: 0.97-1.13; P = 0.28), indicating no significant difference between MMF and CYC in achieving partial renal remission. The level of heterogeneity was reduced to a moderate level (I² = 36%, P = 0.11) after the outlying study was excluded (Figure 9). The funnel plot was used to visually inspect the distribution of studies around the pooled effect estimate and indicated an approximately symmetrical distribution with small studies showing some scatter. This indicates that there is no large study effect or publication bias among the included studies (Figure 10).

Safety Outcomes

**Figure 11:**
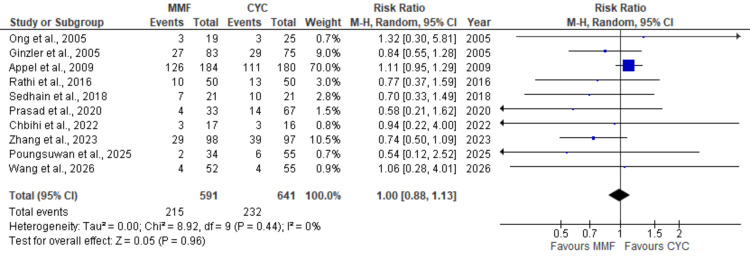
Forest plot of infection MMF: mycophenolate mofetil, CYC: cyclophosphamide, CI: confidence interval

**Figure 12:**
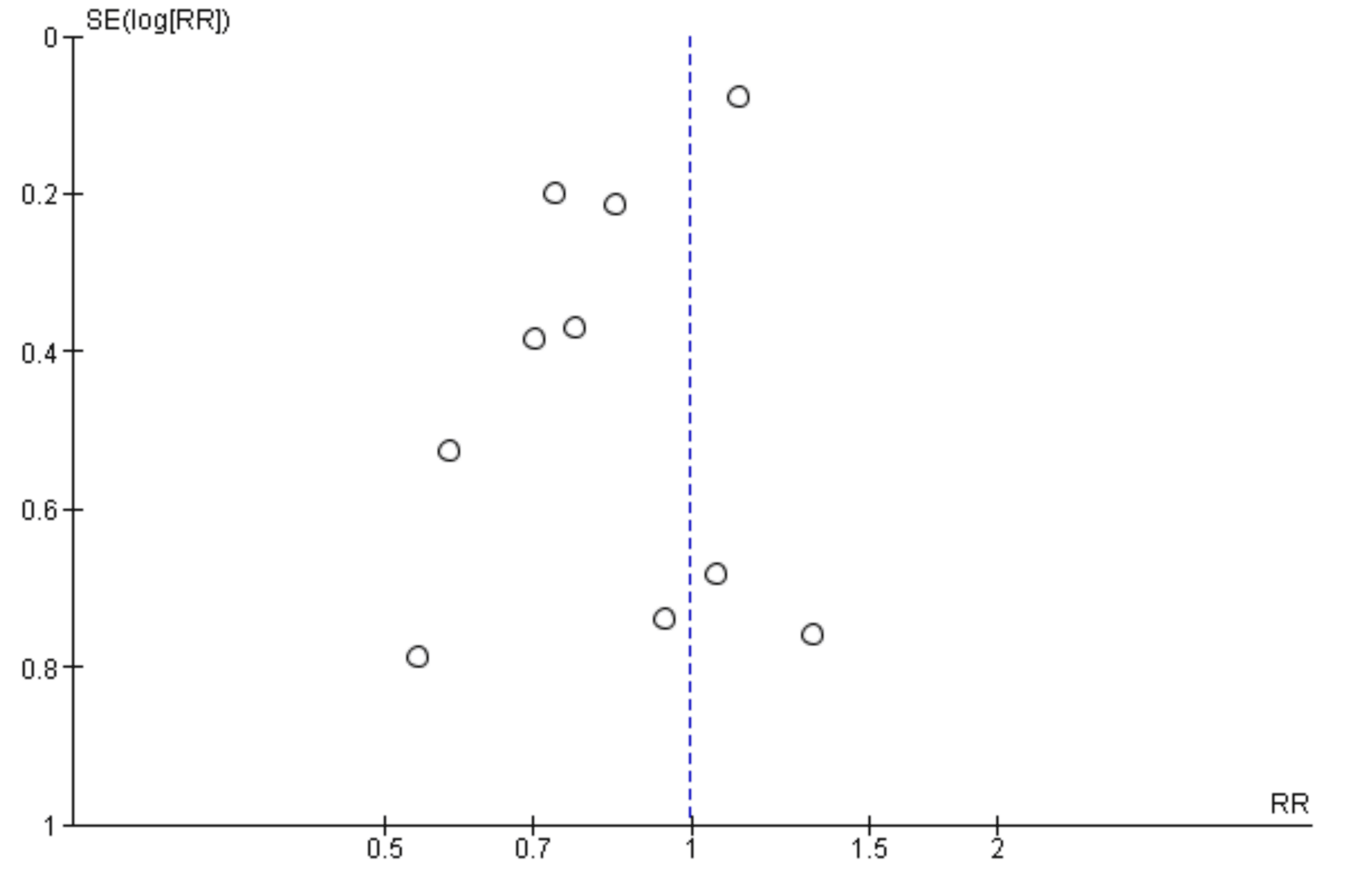
Funnel plot assessing publication bias for infection outcomes

Infection: A pooled analysis of 10 studies [12,22-26,28-31], including 1,232 participants, was used for the second outcome. No statistically significant difference was found between MMF and CYC (RR = 1.00, 95% CI: 0.88-1.13, P = 0.96). There was no significant heterogeneity among the studies (P = 0.44; I² = 0%), indicating consistent findings. In summary, both MMF and CYC appear equally effective for this outcome among patients receiving induction therapy for LN (Figure 11). The funnel plot of the infection outcomes was fairly symmetric, with no significant publication error in the included studies. In most studies, the distribution was fairly even around the overall RR (RR = 1) line, with no noticeable asymmetry or clustering. Additionally, the spread of smaller studies at the lower part of the plot was balanced, further supporting the absence of significant small-study effects. Overall, the funnel plot suggests little evidence of publication bias in the meta-analysis comparing the risk of infection between MMF and CYC (Figure 12).

**Figure 13:**
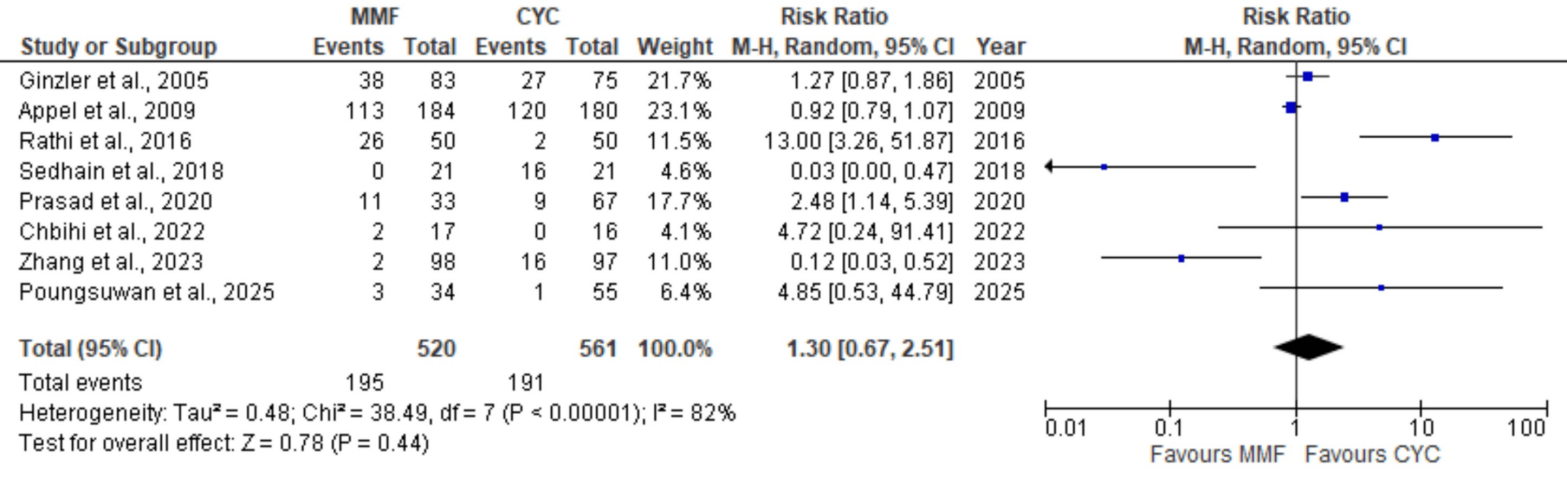
Forest plot of GI adverse events MMF: mycophenolate mofetil, CYC: cyclophosphamide, CI: confidence interval

**Figure 14:**
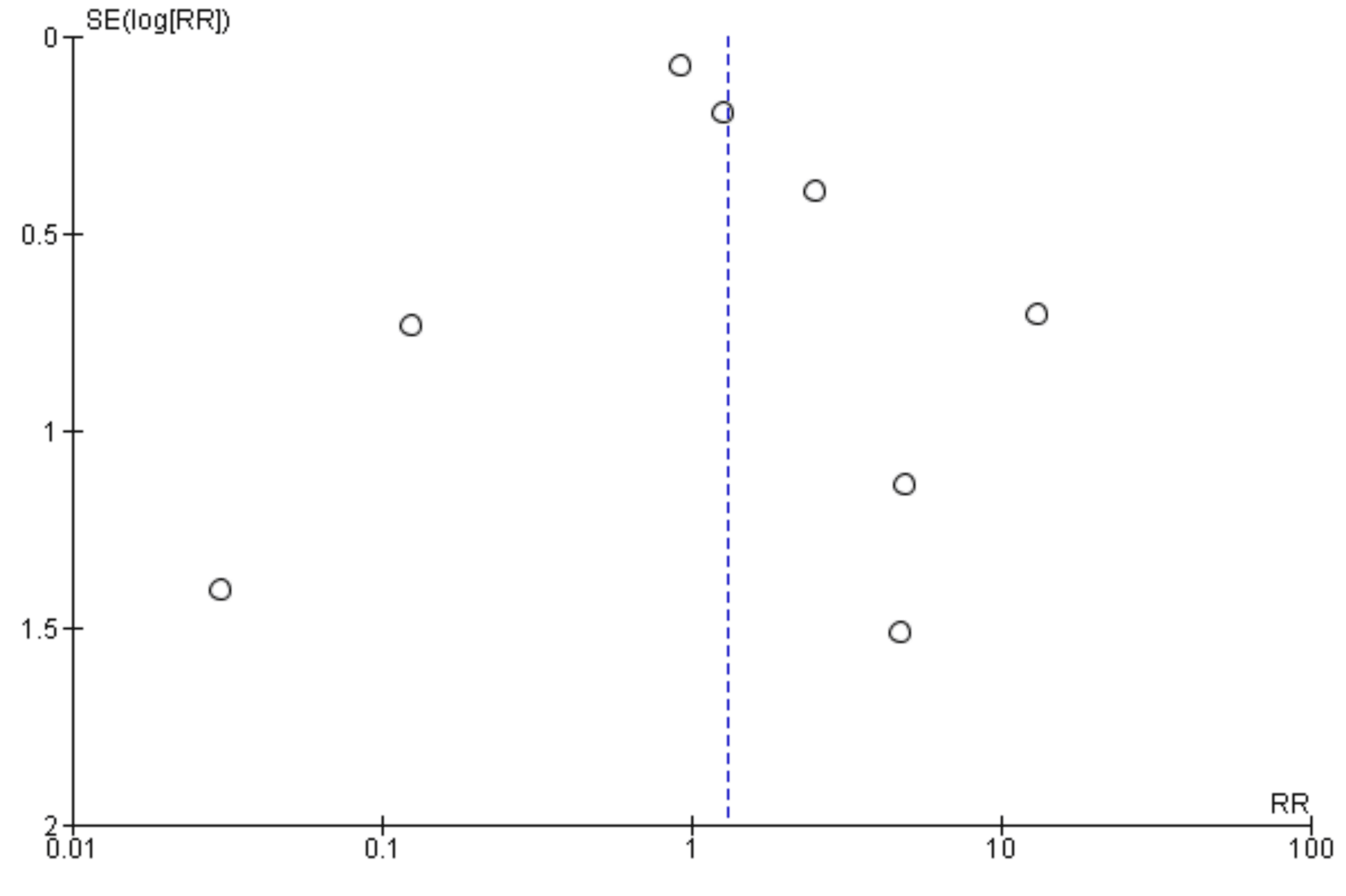
Funnel plot assessing publication bias for GI adverse events GI: gastrointestinal

GI adverse events: The pooled analysis for this outcome included eight studies [12,22,24-26,28-30] with a total of 1,081 participants (MMF = 520; CYC = 561). There was no statistically significant difference between MMF and CYC (RR = 1.30, 95% CI: 0.67-2.51, P = 0.44). The pooled effect estimate favored CYC slightly but did not rule out the null effect, suggesting that the two treatment groups were equally effective for induction therapy in LN. There was significant heterogeneity among the included studies (P < 0.00001; I² = 82%), indicating substantial variation in the studies' findings (Figure 13). The funnel plot was moderately asymmetrical around the pooled effect estimate, which could indicate publication bias or small-study effects. Some small studies exhibited even more extreme treatment effects in both directions relative to the pooled RR line, accounting for possible asymmetry (Figure 14).

Discussion

This meta-analysis demonstrated that MMF was associated with a significantly higher rate of complete renal remission compared with CYC in patients with LN. However, no significant differences were observed between the two therapies with respect to partial renal remission, total renal response, infection risk, or GI adverse events. The findings for complete and partial remission were highly consistent across studies, whereas substantial heterogeneity was observed for total renal response and GI adverse events. Sensitivity analysis for total renal response reduced heterogeneity without altering the overall findings, supporting the robustness of the pooled estimates. Overall, these results suggest that MMF provides comparable efficacy to CYC across most evaluated outcomes while offering a significant advantage in achieving complete renal remission. The findings of the present meta-analysis are broadly consistent with previous reviews by Kamanamool et al., Liu et al., Henderson et al., Palmer et al., and Jiang et al., which reported comparable overall renal response rates between MMF and CYC [13-17]. Similar to those studies, we observed no significant differences in partial remission, total renal response, or infection risk. However, by incorporating recently published studies, our updated analysis demonstrated a statistically significant advantage of MMF in achieving complete renal remission while maintaining a comparable overall safety profile.

Furthermore, a recent systematic review and meta-analysis by Sarkar et al., focusing specifically on childhood-onset LN, also reported comparable efficacy between MMF and CYC, with a potentially more favorable safety profile for MMF [18]. While the analysis by Sarkar et al. was restricted to pediatric patients, the present study included both pediatric and adult populations. It incorporated several recently published studies, thereby providing a broader assessment of the comparative efficacy and safety of MMF and CYC as induction therapies for LN.

MMF was associated with a significantly higher rate of complete renal remission, a finding of particular clinical importance because early and sustained renal remission is strongly associated with improved long-term renal survival and a lower risk of progression to end-stage renal disease in patients with LN [1,7]. This therapeutic benefit may be attributable, at least in part, to MMF's selective inhibition of inosine monophosphate dehydrogenase, which suppresses T- and B-lymphocyte proliferation while maintaining a relatively targeted immunosuppressive effect [1,3]. In contrast, CYC produces broader cytotoxic immunosuppression, which may contribute to its greater toxicity burden. These results are consistent with other landmark trials reporting similar or better renal outcomes with MMF compared with intravenous CYC.

Likewise, recent observational studies in real-world settings have shown that MMF is well-tolerated and achieves favorable remission rates across various ethnic groups [22,24].

No significant differences were observed in partial remission or overall renal response, whereas MMF demonstrated superiority in achieving complete renal remission. These findings suggest that both MMF and CYC remain effective induction agents for managing active renal inflammation in LN. The lack of differences in overall renal response may reflect heterogeneity among the included studies. These results are consistent with previous studies showing that MMF and CYC achieved comparable overall renal remission and that the definitions of remission, the length of follow-up, and the treatment protocol differed between studies, potentially accounting for some of the variation in renal response outcomes [15,18]. Furthermore, significant heterogeneity was observed in the pooled analysis of total renal response, which may be attributable to differences in patient populations, LN classes, pediatric versus adult patients, and CYC regimens. Sensitivity analyses, however, did not change the overall conclusions derived from the pooled estimates and reduced the level of heterogeneity [13,16].

There were no significant differences in infection risk or GI adverse events between MMF and CYC. Comparable infection and mortality rates suggest that both treatments have comparable overall immunosuppressive activity in the induction phase. The GI adverse events were more frequent in the MMF group, but the difference was not statistically significant, and substantial heterogeneity was observed among studies. These results are consistent with previous studies, which found no difference in mortality or the risk of major infections between MMF and intravenous CYC and which reported variation in study definitions of outcomes and follow-up durations [33]. This may reflect variation in MMF dosage, the length of treatment, and the use of other medications containing steroids, as well as differences in reporting of adverse reactions. Similarly, there was a numerical decrease in leukopenia with MMF, although this overall pooled estimate was not statistically significant. The findings also confirm earlier meta-analyses reporting a reduction in the incidence of leukopenia with MMF relative to CYC, although not all studies reported significant differences [14].

Strengths

This meta-analysis has several important strengths. First, it provides an updated synthesis of the available evidence comparing MMF and CYC for induction therapy in LN, incorporating both adult and pediatric populations and including recently published studies not available in earlier reviews. Second, publication bias was assessed using funnel plots for all major outcomes. The funnel plots for complete remission, partial remission, total renal response, and infection demonstrated an approximately symmetrical distribution of studies, suggesting no substantial evidence of publication bias. Furthermore, although significant heterogeneity was observed in the pooled analysis for total renal response (I² = 83%), sensitivity analysis reduced heterogeneity to a moderate level (I² = 36%) without materially changing the overall findings, supporting the robustness and reliability of the pooled estimates. Collectively, these findings strengthen confidence in the validity of the study conclusions.

Limitations

Several limitations should be considered when interpreting the findings of this review. Significant heterogeneity was observed for GI adverse events (I² = 82%), which may reflect differences in MMF dosing, CYC regimens, concomitant corticosteroid therapy, duration of follow-up, and adverse-event reporting methods across studies. Similarly, heterogeneity was observed in the pooled analysis of total renal response, potentially due to differences in remission definitions, follow-up duration, patient populations, LN class distribution, and treatment protocols. Although sensitivity analyses supported the stability of the findings, residual heterogeneity cannot be completely excluded. Additionally, several outcomes were reported in a limited number of studies, reducing the statistical power of certain analyses. Publication bias should also be interpreted with caution because some outcomes were based on fewer than 10 studies. Finally, subgroup analyses according to age, baseline renal function, degree of proteinuria, histological class, ethnicity, and disease severity could not be performed because these variables were either inconsistently reported or unavailable in a poolable format.

Future implications

Future research should focus on large, multicenter, high-quality RCTs with standardized definitions of renal remission, adverse events, and follow-up periods to improve comparability across studies. Studies evaluating long-term outcomes, including renal survival, relapse prevention, progression to chronic kidney disease or end-stage renal disease, cumulative treatment toxicity, fertility outcomes, and health-related quality of life, are particularly needed. Additionally, future investigations should report detailed patient-level characteristics, such as age, baseline renal function, degree of proteinuria, histological class, ethnicity, and disease severity, to facilitate clinically meaningful subgroup analyses. Given the heterogeneity observed in total renal response and GI adverse events, further research is warranted to determine whether treatment effects vary by LN subtype, CYC regimen, MMF dose, ethnicity, or pediatric versus adult populations. Such evidence would help optimize the selection of individualized treatments and further strengthen the evidence base for induction therapy in LN.

## Conclusions

This meta-analysis demonstrated that MMF was associated with a significantly higher rate of complete renal remission than CYC during induction therapy for LN. No significant differences were observed between the two therapies in partial renal remission, total renal response, infection risk, or GI adverse events. Although substantial heterogeneity was observed for total renal response and GI adverse events, sensitivity analysis supported the robustness of the overall findings. Taken together, these results suggest that MMF provides an effective alternative to CYC for induction therapy in LN and may offer advantages in achieving complete renal remission while maintaining a comparable overall safety profile.

## Appendices

**Table 6:** Supplementary materials

PubMed = 663	Embase = 3,490	Scopus = 1600	Ccohrane = 567
("Lupus Nephritis"[MeSH Terms] OR "Lupus Nephritis"[All Fields] OR "systemic lupus erythematosus nephritis"[All Fields] OR "SLE nephritis"[All Fields]) AND ("Mycophenolic Acid"[MeSH Terms] OR "mycophenolate mofetil"[All Fields] OR "MMF"[All Fields] OR ("mycophenolate"[All Fields] OR "mycophenolates"[All Fields] OR "mycophenolic"[All Fields])) AND ("Cyclophosphamide"[MeSH Terms] OR ("Cyclophosphamide"[Supplementary Concept] OR "Cyclophosphamide"[All Fields] OR "cyclophosphamid"[All Fields] OR "Cyclophosphamide"[MeSH Terms] OR "cyclophosphamide s"[All Fields] OR "cyclophosphamides"[All Fields]) OR "CYC"[All Fields])	('lupus nephritis'/exp OR 'lupus nephritis' OR 'systemic lupus erythematosus nephritis' OR 'sle nephritis') AND ('mycophenolate mofetil'/exp OR 'mycophenolate mofetil' OR mmf OR mycophenolate) AND ('cyclophosphamide'/exp OR 'cyclophosphamide' OR cyc)	( TITLE-ABS-KEY ( "lupus nephritis" OR "systemic lupus erythematosus nephritis" OR "SLE nephritis" ) AND TITLE-ABS-KEY ( "mycophenolate mofetil" OR MMF OR mycophenolate ) AND TITLE-ABS-KEY ( cyclophosphamide OR CYC ) )	#1 ("lupus nephritis" OR "systemic lupus erythematosus nephritis" OR "SLE nephritis") #2 ("mycophenolate mofetil" OR MMF OR mycophenolate) #3 (cyclophosphamide OR CYC) #4 #1 AND #2 AND #3
